# PP19 Initial Exploration Of Generative Artificial Intelligence: Identified Tasks, Team Perspectives, And Early Implementation Insights

**DOI:** 10.1017/S0266462325102390

**Published:** 2025-12-29

**Authors:** Maria Dolors Estrada Sabadell, Joan Segur-Ferrer, Roland Pastells-Peiró, Jessica Ruiz-Baena, Rosa Maria Vivanco-Hidalgo

## Abstract

**Introduction:**

Artificial intelligence (AI) in health technology assessment (HTA) report development addresses three needs: accelerating processes via automation, standardizing tools for quality and consistency, and training to responsibly integrate generative AI. Conducted at the Agency for Health Quality and Assessment of Catalonia (AQuAS), this study identified tasks that generative AI can enhance and evaluated the team’s perceptions, knowledge, attitudes, and experience with these tools.

**Methods:**

A review of technical literature and an exploration of leading tools, including ChatGPT and Copilot, were conducted to identify relevant functionalities and their potential applications in HTA. Additionally, an online survey was designed to assess the team’s knowledge and use of AI with four sections: (i) team characteristics; (ii) knowledge and use of generative AI; (iii) practical applications and perceived utility in specific tasks; and (iv) perspectives on potential and adoption barriers. Created using Microsoft Forms, the survey was distributed in October 2024.

**Results:**

An initial best practices guide was developed, detailing how to interact with generative AI tools, key elements for integration, and practical application examples, including training materials to mitigate risks. The survey achieved a 100 percent response rate, with participation from the entire evaluation team (n=14) and administrative support (n=1). Of respondents, 53 percent reported intermediate knowledge, 80 percent use these tools in daily tasks, and 41 percent have used them for over a year. Among users (n=12), key tasks included text drafting (92%), information retrieval (83%), and PDF reading (67%). Participants reported high satisfaction, and non-users expressed willingness to adopt these tools.

**Conclusions:**

Generative AI showed great potential to enhance efficiency and quality in report development at AQuAS. High satisfaction among current users and a willingness to adopt it by non-users supported its implementation. The initial best practices guide and ongoing training will be essential to standardize the use of AI and maximize its impact.

